# Molecular identification of microorganisms associated with the brine shrimp *Artemia franciscana*

**DOI:** 10.1186/2046-9063-9-7

**Published:** 2013-03-08

**Authors:** Misty R Riddle, Bonnie K Baxter, Brian J Avery

**Affiliations:** 1 Department of Biology and Great Salt Lake Institute, Westminster College, 1840 South 1300 East, Salt Lake City, UT 84105, USA; 2 Present address: Department of Molecular, Cellular and Developmental Biology, UC Santa Barbara, Santa Barbara, CA 93106, USA

## Abstract

**Background:**

Prior research on the microorganisms associated with the brine shrimp, *Artemia franciscana*, has mainly been limited to culture-based identification techniques or feeding studies for aquaculture. Our objective was to identify bacteria and archaea associated with *Artemia* adults and encysted embryos to understand the role of microbes in the *Artemia* life cycle and, therefore, their importance in a hypersaline food chain.

**Results:**

We used small subunit (SSU) 16S ribosomal RNA gene sequencing to identify bacteria and archaea associated with adults and encysted *Artemia* embryos from one of their natural environments – Great Salt Lake (GSL), Utah, USA. We found that bacterial sequences most closely related to the genera *Halomonas* and *Vibrio* were commonly extracted from GSL adult *Artemia*, while bacterial sequences most similar to the genera *Halomonas*, *Psychroflexus* and *Alkalilimnicola* dominate in GSL water. Encysted embryos (cysts) yielded bacterial sequences from the genera *Idiomarina* and *Salinivibrio*, which were absent from adults and water. Common archaeal sequences in adults were most closely related to the genera *Haloterrigena* and *Haloarcula*, while all of the archaeal sequences from GSL water were most similar to the genus *Halogeometricum*. Cyst derived archaeal sequences were most closely related to the genera *Halorubrum* and *Haloarcula*.

**Conclusions:**

In addition to identifying microbial rRNA sequences that are specific to different stages of the *Artemia* life cycle, we observed striking differences in the sequences associated with the adult *Artemia* population in samples collected from GSL at different times and locations. While our study was limited in scope and the sample was small, our findings provide a foundation for future research into how the bacteria and archaea associated with *Artemia* influence the *Artemia* life cycle, and GSL food web.

## Background

*Artemia franciscana*, the brine shrimp that inhabits many hypersaline environments including Great Salt Lake (GSL) in Utah, is an important food source for migrating birds and is used as fish food in aquaculture [1,2]. Most studies concerning *Artemia* and microbes are related to industrial production of *Artemia*[3]. These studies indicate that microorganisms may be involved in the *Artemia* life cycle as a food source [4] and for protection from pathogenic bacteria [5]. Using electron microscopy, intracellular symbiotic microbes, identified as spirochetes, have been detected in the epithelial cells of the midgut in GSL *Artemia*[6]. Little is known, however, about the interaction between brine shrimp and the microbial community in their natural ecosystem. In this study, we present bacterial and archaeal 16S rRNA gene sequences isolated from *Artemia* adults and dormant encysted *Artemia* embryos (cysts) from GSL.

We focused our efforts on Great Salt Lake (GSL) in Utah, which is one of the largest hypersaline lakes in the world. *Artemia* are abundant inhabitants of the moderately saline South Arm of GSL (~11-15% w/v sodium chloride equivalent in recent years), which is separated from the much saltier North Arm by a 50-year old railroad causeway [7]. The lake has a relatively simple food web involving roughly 250 million migrating birds that eat the two invertebrate inhabitants of GSL – the brine fly (*Ephedra* spp.) and *Artemia*[1]. GSL *Artemia* are thought to be supported by a rich phytoplankton community dominated by the species *Dunaliella viridis*[8] yet little is known regarding the bacteria and archaea that live in association with GSL *Artemia* or how these microbes contribute to the GSL food web. Previous studies that have identified microorganisms from adult brine shrimp in natural environments or from commercially harvested cysts have used culture techniques to identify a range of gram negative and gram positive bacterial species [9-16], with one exception that examined bacterial Ribosomal RNA (rRNA) gene sequences associated with *Artemia* from salterns in Isreal [17]. No archaeal species have been identified. Since microbes from aquatic environments are often difficult to culture or are unculturable in a laboratory setting [18,19], the results of these previous cultured-based studies may be dominated by microbes that grew more successfully in culture rather than represent a sample of the natural populations [20,21].

rRNA gene sequence has been used as a culture-independent method to identify bacteria and archaea that inhabit natural environments from oceans to deserts, inhospitable places such as rocks and salt crystals, as well as microbes that are associated with other living organisms [22-26]. While culture-independent techniques may have their own biases [27,28], we used this method to identify a different set of microorganisms present in *Artemia* samples than previously reported in the literature (with the exception of members of the genus *Vibrio* identified by several studies and members of the genera *Halomonas, Salinivibrio,* and *Roseovarius*[17]), including the first report of archaea associated with *Artemia*.

Our initial step toward understanding how microorganisms affect the *Artemia* life cycle and surrounding food web was to identify microbial 16S rRNA genes associated with adult *Artemia*. We then compared these data to sequences from GSL water to test the hypothesis that *Artemia* harbor some microbes at much higher concentrations than the surrounding water. We also hypothesized that some microbes may be specific to encysted embryos, and that there may be some microbes in common between cysts and adults. In order to test this hypothesis, we identified microbial 16S rRNA sequences associated with *Artemia* cysts from GSL and compared them to the sequences from adults and GSL water. And finally, we hypothesized that microbes found in association with GSL *Artemia* may also be found in populations from other hypersaline ecosystems. Therefore, we expected to find similarities in the 16S rRNA gene sequences that we isolated from GSL *Artemia* cysts and *Artemia* cysts from the “San Francisco Bay” (SFB) strain harvested from another location.

## Results

### Sequences from adult *Artemia*

We collected 75 sequences from GSL adult *Artemia*-derived clones, 37 were amplified with the bacterial primer set (see Methods and Table 1A) and 48 with the archaeal primer set (see Methods and Table 2A). The bacterial sequences represent seven different genera based on RDP classification: *Vibrio, Halolactibacillus, Halomonas, Roseovarius, Lutibacter, Alkalilimnicola,* and *Caulobacter* (Table 1A). Based on BLAST analysis, six of the eight distinct sequences most closely matched sequences isolated from uncultured microbes. The results of RDP Classifier analysis indicated that the archaeal sequences represent five genera: *Haloterrigena, Haloarcula, Natronobacterium, Halogeometricum,* and *Halovivax*, and according to BLAST analysis three of these groups most closely matched uncultured clones (Table 2A). The most abundant archaeal sequence isolated from adults (AAC1) was most identical to *Haloterrigena limicola* by BLAST. It represents over half of the adult derived clones and was not found in our water or encysted *Artemia* embryo samples. However, we did isolate a single sequence (GAU1) from GSL cysts that was classified as the same genus as AAC1 but matched *Haloterrigena saccharevitans* by BLAST, and when aligned with AAC1 was only 93% identical so it was considered to represent a different organism.

**Table 1:** Phylogenetic affiliations of the uncultured bacteria based on 16S rDNA analysis

**Sequence**	**Clones**	**Closest BLASTn match to NCBI nr database**	**Identities (%)**	**Genus [confidence value]**	**Genbank Accession**
**A. Sequences obtained from GSL Adult *Artemia***					
ABC1	9	DQ068937.1 *Vibrio metschnikovii*	627/631 (99)	*Vibrio*[100%]	KC696895-KC696903
ABC2	5	AB362696.1 *Halolactibacillus halophilus*	615/631 (97)	*Halolactibacillus*[82%]	KC696904-KC696906
ABC3	15	DQ351910.1 Uncultured bacterium clone JH-WH17	592/603 (98)	*Halomonas*[100%]	KC696872-KC696886
ABC4	4	AM691100.1 Uncultured “Rhodobacteraceae”	582/589 (98)	*Roseovarius*[100%]	KC696887-KC696892
ABU1	1	EU245085.1 Uncultured organism clone MAT-CR-H1-G02	629/661 (95)	*Alkalilimnicola*[16%]	KC696869
ABU4	1	AM420114.1 Uncultured alpha proteobacterium	532/532 (100)	*Caulobacter*[98%]	KC696870
ABU6	1	DQ396185.1 Uncultured organism clone ctg_NISAA81	581/587 (98)	*Halomonas*[100%]	KC696871
ABU7	1	DQ154838.1 Uncultured bacterium clone GN01-0.012	513/534 (96)	*Lutibacter* [15%]	KC696893-KC696894
**B. Sequences obtained from GSL *Artemia *cysts**					
GBRC1	14	DQ462298.1 Uncultured bacterium clone e41	525/531 (98)	*Idiomarina*[100%]	KC703296-KC703308
GBRC2	4	X95527.1 SCRR701T *S. costicola* (strain NCIMB 701-T)	496/497 (99)	*Salinivibrio*[100%]	KC70329-KC703295
GBRU1	1	EU287134.1 Uncultured bacterium clone P13-41	406/430 (94)	*Marinimicrobium*[96%]	KC703293
GBRU2	1	EF190068.1 Uncultured *Psychroflexus* sp. clone GSX1	543/587 (92)	*Gramella*[41%]	KC703291
GBRU3	1	DQ157009.1 “Marinobacter haloterrigenus” strain FP2.5	488/518 (94)	*Marinobacter*[54%]	KC703292
**C. Sequences obtained from SFB strain *Artemia *cysts**					
SBR1	15	X95527.1 SCRR701T *S. costicola* (strain NCIMB 701-T)	618/622 (99)	*Salinivibrio*[100%]	KC696910-KC696930
SBR2	3	EF177666.1 *Idiomarina* sp. Y24	564/565 (99)	*Idiomarina*[100%]	KC696931-KC696933
SBU1	1	EU135665.1 *Lysobacter* sp. YIM C734	566/596 (94)	*Lysobacter*[75%]	KC696909
SBU2	1	AF505746.1 Gamma proteobacterium UMB21A	562/566 (99)	*Psychrobacter*[100%]	KC696907
SBU3	1	AM084035.1 *Acidovorax* sp. R-25076	564/564 (100)	*Acidovorax*[100%]	KC696908
**D. Sequences obtained from GSL water**					
HBRC2	4	AM691089.1 Uncultured *Psychroflexus* sp. isolate EG26	554/559 (99)	*Psychroflexus*[100%]	KC696938-KC696940, KC696944-KC696945
HBRC3	4	AM691089.1 Uncultured *Psychroflexus* sp. isolate EG26	531/546 (97)	*Psychroflexus*[99%]	KC696935-KC696937, KC696941-KC696943
HBRC4	9	EF554887.1 *Halomonas* sp. G27	512/527 (97)	*Halomonas*[100%]	KC696946-KC696952, KC696963-KC696966
HBRC5	7	AY862776.2 Uncultured proteobacterium clone At18AugB10	492/492 (100)	*Alkalilimnicola*[74%]	KC696953-KC696962
HBUN1	1	AY862797.2 Uncultured actinobacterium clone At12OctB10	413/418 (98)	*Agrococcus* [59%]	KC696934

Sequence names, number of clones, BLASTn matches with percent identity, and genus assignment according to RDP Classifier with confidence values in brackets are listed for all of the bacterial sequences. Part A includes sequences derived from GSL *Artemia* adults, Part B is sequences from GSL *Artemia* cysts, Part C is sequences from SFB strain *Artemia* cysts, and Part D shows sequences from GSL water.

**Table 2:** Phylogenetic affiliations of the uncultured archaea based on 16S rDNA analysis

**Sequence**	**Clones**	**Closest BLASTn match to NCBI nr database**	**Identities (%)**	**Genus [confidence value]**	**Genbank Accession**
**A. Archaeal sequences obtained from GSL adult *Artemia***					
AAC1	26	DQ367241.1 *Haloterrigena limicola* strain AX-7	481/485 (99)	*Haloterrigena*[100%]	KC696970-KC696994
AAC3	17	EF645686.1 *Haloarcula japonica* strain JCM7785	502/504 (99)	*Haloarcula*[100%]	KC696995-KC697011
AAC4	2	AJ969886.1 Uncultured archaeon, clone ss_014	330/354 (93)	*Natronobacterium*[28%]	KC697012-KC697013
AAC5	2	AJ969890.1 Uncultured archaeon, clone ss_010a	498/503 (99)	*Halogeometricum*[53%]	KC696968-KC696969
AAU9	1	EF690637.1 Uncultured haloarchaeon clone TX4CA_82	497/520 (95)	*Halovivax*[57%]	KC696967
**B. Archaeal sequences obtained from GSL *Artemia *cysts**					
GAC1	10	AY510707.1 *Halorubrum xinjiangense*	522/524 (99)	*Halorubrum*[100%]	KC697020-KC697029
GAC2	8	EF645686.1 *Haloarcula japonica* strain JCM7785	522/524 (99)	*Haloarcula*[100%]	KC697030-KC697037
GAC3	6	AM269467.1 *Halovivax ruber* type strain XH-70 T	507/519 (97)	*Halovivax*[89%]	KC697015-KC697019
GAU1	1	AY820137.2 *Haloterrigena saccharevitans* strain AB14	499/524 (95)	*Haloterrigena*[35%]	KC697014
**C. Archaeal sequences obtained from SFB strain *Artemia *cysts**					
SAC1	2	EF468473.1 *Halorubrum tebenquichense* strain JCM12290	521/524 (99)	*Halorubrum*[100%]	KC697047-KC697048
SAC2	3	AY820137.2 *Haloterrigena saccharevitans* strain AB14	518/524 (99)	*Haloterrigena*[75%]	KC697049-KC697051
SAC4	4	AY570917.1 “Natrinema ajinwuensis” strain AJ12	499/524 (95)	*Natronorubrum*[81%]	KC697041-KC697044
SAC5	2	DQ103672.1 Uncultured euryarchaeote clone ArcH05	479/509 (94)	*Halogeometricum*[26%]	KC697045-KC697046
SAU1	1	AJ969890.1 Uncultured archaeon clone ss_010a	378/409 (99)	*Halogeometricum*[50%]	KC697039
SAU4	1	DQ431096.1 Uncultured archaeon clone A10	458/478 (96)	*Halogeometricum*[70%]	KC697038
SAU5	1	EF632847.1 Uncultured archaeon clone Hua-w-29	300/302 (99)	*Halorubrum*[100%]	KC697040
SAU7	1	EF077641.1 *“*Halorubrum alimentarium” strain B43	180/181 (99)	*Halorubrum*[97%]	unavailable
**D. Archaeal sequences obtained from GSL water**					
HAC1	29	AF196290.1 Archaeon JDS-1	503/504 (99)	*Halogeometricum*[49%]	KC697052-KC697086

Sequence names, number of clones, BLASTn matches with percent identity, genus assignment according to RDP Classifier with confidence values in brackets, and Genbank accession numbers are listed for all of the archaeal sequences. Part A includes sequences derived from GSL *Artemia* adults, Part B is sequences from GSL *Artemia* cysts, Part C is sequences from SFB strain *Artemia* cysts, and Part D shows sequences from GSL water.

**Figure 1:**
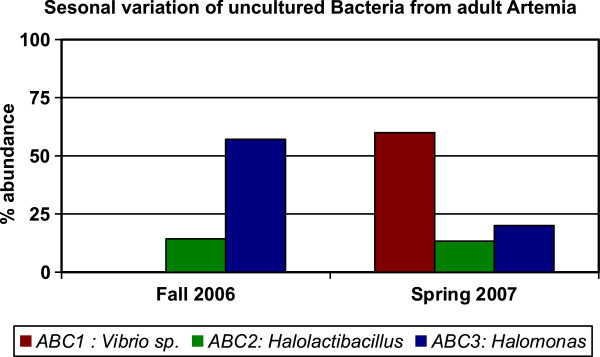
**Variation of bacterial 16S rRNA gene sequences from different GSL adult *Artemia *samples.** Percent abundance of a sequence was calculated by dividing the number of clones with that sequence in the season being analyzed by the total number of all clones from that season. Only contigs that were constructed from five or more total clones were included in the analysis. The genus that each sequence most closely matched is shown for reference. ABC1 was only present in the Spring 2007/DWR3 sample.

Of the bacterial clones amplified from adult *Artemia,* 21 clones were from the sample collected in the Fall of 2006 at Black Rock and 15 clones were from the sample collected in Spring 2007 at DWR3. The number of sequences that made up some contigs varied between these two samples. For example, contig ABC1 consisted of clones that were all from the Spring 2007/DWR3 sample, suggesting that this sequence was much more abundant at that time and location. This sequence was classified as being from the genus *Vibrio* according to RDP Classifier and BLAST. Sequence ABC2 was isolated with similar frequency in both samples, while ABC3 (identified as *Halomonas* by RDP and BLAST) was more abundant in the adult *Artemia* from the Fall 2006/Black Rock sample*.* Percent abundance of clones from each sample was determined for each sequence and is graphed in Figure 1. We found a significant difference in the bacterial sequence distribution between the two samples (p < .001).

**Figure 2:**
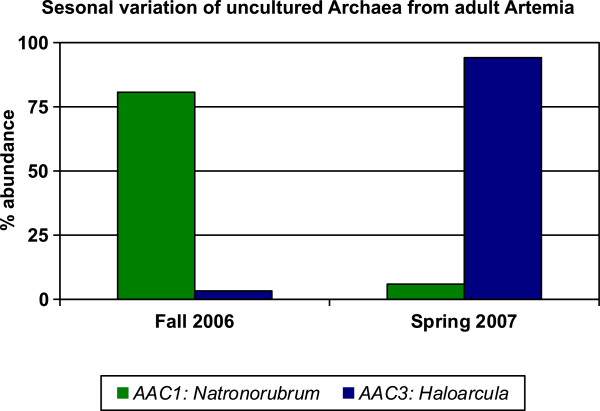
**Variation of archaeal 16S rRNA gene sequences isolated from different GSL adult *Artemia *samples.** Percent abundance of a sequence was calculated by dividing the number of clones with that sequence in the season being analyzed by the total number of all clones from that season. Only contigs that were constructed from five or more total clones were included in the analysis. The genus that each sequence most closely matched is shown for reference.

The archaeal data from adults were also analyzed for variation between samples as described above (Figure 2). A total of 31 clones from the Fall 2006/Black Rock sample and 17 clones from the Spring 2007/DWR3 sample were sequenced. The number of AAC1 (*Haloterrigena*) clones from the Fall 2006/Black Rock sample was higher than the number of contributing clones from the Spring 2007/DWR3 sample. This suggests that sequence AAC1 was more abundant in the Fall 2006/Black Rock sample*.* Sequence AAC3 (*Haloarcula*) was constructed of clones only from the Spring 2007/DWR3 sample. There was a significant difference in sequence distribution between these two samples (p < .001).

### Sequences from encysted *Artemia* embryos

To continue our search for microbes associated with *Artemia*, we amplified bacterial and archaeal 16S rRNA genes from encysted *Artemia* embryos from two different sources of commercially harvested dry cysts – one identified as “San Francisco Bay” (SFB) strain and one from Great Salt Lake (GSL) (see Methods). A total of 45 clones isolated from GSL cysts were sequenced, 20 from the bacterial primer set (Table 1B) and 25 from the archaeal set (Table 2B). A total of 36 clones isolated from SFB strain cysts were sequenced, 21 from the bacterial primer set (Table 1C) and 15 from the archaeal primer set (Table 2C). Some of the SFB strain cyst derived sequences were classified as the same genus by RDP, yet were dissimilar enough to have different closest match sequences according to BLAST analysis so were considered separately.

Sequences that were most identical to *Salinivibrio costicola* by BLAST were found in both SFB strain and GSL cysts. When the sequences (GBRC2 and SBR1) were compared they were 99% identical which indicates that they are likely to be the same species or very closely related. Also, sequences classified as *Idiomarina* were found in both SFB strain and GSL cysts (GBRC1 and SBR2, 99% identical).

The most abundant archaeal sequence found in our GSL cyst derived clones (Table 2B, GAC1, 40% of sequences) was most identical to *Halorubrum xinjiangense* by BLAST. Several different sequences classified as *Halorubrum* by RDP were also isolated from SFB strain cysts at low abundances. When the sequence of GAC1 was compared to either SAU5 or SAU7 each pair was 97% identical so they were considered separately. No sequences closely related to the genus *Halorubrum* were found in GSL water or adult samples. Other sequences that were isolated from both SFB strain and GSL cysts were GAU1 from GSL cysts and SAC2 from SFB strain cysts which both had *Haloterrigena saccharevitans* as their top BLAST match (Tables 2B and 2C). However, they were only 95% identical when compared to each other.

Some sequences that were abundant in GSL cysts were not found in SFB strain cyst-derived clones. For example, sequence GAC3, which was most closely related to *Halovivax ruber* by BLAST, was 24% of clone sequences from GSL cysts and was not found in clones from SFB strain cysts (Tables 2B and 2C). The single sequence AAU9 from adults was also classified as *Halovivax* by RDP, but it was only 93% identical to GAC3 and was considered separately. Other sequences of low abundance that were isolated from either SFB strain or GSL cysts are listed in Tables 1 and 2.

### Sequences from GSL water samples

We also derived microbial 16S rRNA gene sequences from GSL water samples to compare the external microbial environment to the microbes we found within adult and encysted *Artemia*. A total of 54 sequences were obtained from two samples of GSL water from Black Rock, 25 sequences with the bacterial primer set (Table 1D) and 29 from the archaeal primer set (Table 2D). The bacterial genera found in GSL water derived sequences were: *Psychroflexus*, *Halomonas* (also in adults), *Alkalilimnicola*, and *Agrococcus*. The abundance of these sequences did not vary between the samples. Sequence HBRC2 and HBRC3 from GSL water were most identical to *Psychroflexus* sp. isolate EG26 by BLAST but were only 96% identical to each other so they were included separately.

All of the 29 archaeal clones that were amplified from GSL water collected at Black Rock formed a single contig (HAC1, Table 2D). This sequence was classified as Archaeon JDS-1 (AF196290) by BLAST and as a member of the genus *Halogeometricum* by RDP. This sequence was not identified in *Artemia* cysts or adults and, in contrast, the archaeal sequences from adults collected at the same location represented five different genera. One contig of two sequences (AAC5, 4% of sample) from adults was also classified as *Halogeometricum* but had a different top BLAST match and was only 86% identical to the sequences from water. While neither sample was exhaustive and the number of archaeal clones sequenced from GSL water was fewer than adults, the difference between these two samples was statistically significant (p < .001).

## Discussion

### 16S rRNA gene sequences isolated from adult *Artemia*

Microbes have been shown to affect the growth and survival of *Artemia* in the laboratory [4,5,14]. However, little is known about the bacteria and archaea associated with *Artemia* in natural ecosystems or how these microbes affect the surrounding food web. To begin to understand this relationship, we surveyed 16S rRNA gene sequences to identify microbial populations specifically associated with GSL *Artemia*. We have successfully identified microbes distinct from those found in GSL water and not previously reported in the literature as being associated with *Artemia*. In addition, we isolated archaeal sequences during our analysis of *Artemia*-associated microbes, which have not been previously reported.

Clone sequence AAC1 (identified as *Haloterrigena limicola* by BLAST) was abundant in adult *Artemia* and not in GSL water samples or cysts, which leads us to hypothesize that the organism it represents could be an *Artemia* symbiont or is at least concentrated in or on adult *Artemia*. There are examples of microbes that are tightly associated with the surface of crustaceans, referred to as epibiotic or episymbiotic organisms [29,30]. We cannot rule out the effects of our relatively small sample size and limited sampling locations as possible alternative explanations for the absence of sequences such as AAC1 in our water and cyst samples, but no sequences assigned to that genus were found by Meuser et al. (2013) in their study of the microbial communities that inhabit the water column at the DWR3 site, which included 307 archaeal sequences [31].

While there were no bacterial sequences identified in both adults and cysts, there were several shared archaeal sequences. For example, AAC3 in adult *Artemia* and GAC2 in GSL *Artemia* cysts were both 99% identical to *Haloarcula japonica* strain JCM7785 by BLAST and are found in similar abundance in both adults and cysts. From our data we cannot determine if the microbes found in adults and cysts are directly transmitted from mother to cyst. We plan to investigate whether some of the microbes we have identified are vertically transmitted by performing *in situ* hybridization on developing *Artemia* with microbe specific probes [32-34]. No sequences assigned to the genus *Haloarcula* were found in our water samples, and a single sequence assigned to *Haloarcula* was found by Meuser et al. (2013) at DWR3 out of a total of 307 [31].

We identified several similar bacterial sequences from both the adult and water derived 16S sequences (ABU1 and HBRC5, ABC3 and HBRC4). ABC3 and HBRC4 were both classified as *Halomonas* by RDP but are most identical to different species when compared to the database with BLAST and were only 96% identical when directly compared to each other. Members of the genus *Halomonas* have been found in other hypersaline environments using culture dependent and independent methods [20,21,35]. Sequences classified as being from the genus *Halomonas* have also been reported in a study of bacterial rRNA sequences associated with *Artemia* from Israeli salterns [17] and from GSL water at very low percentages (8 sequences of 332 total) at intermittent depths in the water column [31]. These observations are consistent with our finding of sequences identified as *Halomonas* in both our adult *Artemia* and water samples.

The sequences ABU1 and HBRC5, which were classified as *Alkalilimnicola* by RDP, were also found in both GSL adults and water*.* The species *Alkalilimnicola* has been isolated from hypersaline environments such as Mono Lake in CA, USA [36], and sequences from this genus are present at very low abundance at 0 m depth in the GSL water column of the DWR3 site [31] but have not been previously isolated from *Artemia*. It is not surprising that there is some overlap between the sequences found in adults and GSL water since *Artemia* feed by funnelling water into their mouths and filtering out microorganisms using special mouth parts, but our data do not address the relationship between these microbes and *Artemia*.

While we identified some similar bacterial sequences in our adult- and water-derived samples, there were also notable differences. The second most abundant sequence found in the GSL adult sample was most identical to *Vibrio metschnikovii* (ABC1, 24% of sample)*.* This species was not found in our water-derived sequences. Similarly, a single clone also matching this same species by BLAST was the only member of the genus *Vibrio* recovered by Meuser et al., out of a total of 332 bacterial sequences anaylzed [31]. Vibrios have been shown to be associated with *Artemia* in previous studies using culture techniques [10,12-14,37] and may be significant to the *Artemia* life cycle as a symbiont or pathogen. Vibrios are often found in association with eukaryotes with relationships ranging from mutualistic to pathogenic [38], and *Vibrio metschnikovii* has been found in association with marine invertebrates [39].

It is also worth noting that we found sequences specific to the water samples. For example HBRC2 and HBRC3 were the only sequences identified by BLAST and RDP Classifier as belonging to the genus *Psychroflexus*. Sequences assigned to this genus were also identified in GSL water by Meuser et al. (2013) [31], and were not present in any of our other samples.

Although the microbes we have identified may be only a subset of the population, we noticed a dramatic change in microbial sequences isolated from adult *Artemia* collected during the Fall of 2006 at Black Rock as compared to *Artemia* collected in the Spring of 2007 at the DWR3 site (p < .001, Figures 1 and 2). While we did not measure salinity or temperature during our collections, spatial and seasonal variation of various GSL conditions are well documented. The salinity [40,41], surface temperature [42] and chlorophyll concentration [43] all vary with the season, as does the brine shrimp population and the ratio of live births to cysts [44]. There are also examples of differences in the composition of microbial communities under different conditions from various other environments, for example, other lakes [45,46], marine environments [46,47], and soil [48] that are consistent with our preliminary analysis. While the differences we have found appear striking, it is possible that they were an artifact due to some unknown variable in our collection, PCR, or other methods. To further explore this variation we could collect more systematic samples of water, adults and cysts from the same location at the same time and analyze the abundance of amplified microbial genes over many seasons and in different locations in the South Arm of GSL.

### 16S rRNA gene sequences isolated from encysted *Artemia* embryos

We hypothesized that cysts would contain a different population of microbes than adults or water due to their unique, desiccated microenvironment. We also became curious as to how many microbes would be shared among cysts from different environments, which led us to compare sequences from the SFB strain and GSL encysted *Artemia* embryos. Our data support this hypothesis since we found cyst specific sequences such as GAC1 (*Halorubrum*), GBRC2/SBR1 (*Salinivibrio*) and GBRC1/SBR2 (*Idiomarina*) that were not in our adult or water samples from GSL. Sequences from *Idiomarina* were absent from the GSL water column samples of Meuser et al. (2013) and there were only 2 *Salinivibrio* sequences out of 332 total [31]. The proportion of GBRC2/SBR1 (*Salinivibrio*) and GBRC1/SBR2 (*Idiomarina*) varied between the cyst samples. Also, a single experiment to culture microbes from GSL cysts produced several colonies that had 16S rRNA gene sequences that clustered with GBRC1/SBR2 (data not shown) indicating that these microbes were associated with these GSL cysts.

Our data show that there were similarities and differences in the microbes associated with *Artemia* cysts from different sources. Similarities between the two samples could be an indicator of species that are important to the *Artemia* life cycle and can persist even in the desiccated cyst environment. Differences could indicate that the microbes associated with brine shrimp are influenced by the surrounding environments and current abiotic conditions. The lack of overlap of archaeal and bacterial sequences between the GSL cyst and water samples, may reflect the dramatic difference in conditions between GSL water and cyst internal environment.

There are, however, some limitations to our work with *Artemia* cysts. We purchased dry commercial cysts rather than collecting cysts from GSL or SFB. The GSL cysts come from an unknown site in the south arm of GSL and the SFB strain cysts may have come from any of several locations around the world where SFB strain or “type” cysts can be found inoculated into evaporation ponds, which limits our ability to draw firm conclusions about a link between the environment from which the cysts were harvested and the microbes they harbor. Also, commercially harvested cysts are often processed and stored under various conditions and this could affect the microbes found in and on them (see also Methods).

To address these limitations and expand our study, we propose a more comprehensive study where water, adults, and cysts would be collected from the same location at the same time. Samples could be taken at different times of the year, such as spring, summer, and winter, although cysts may be limiting at certain times of the year. This more comprehensive sampling approach would allow for more direct comparisons to better understand any life cycle, seasonal, or geographic variation in the microbial community. Also, cysts could be decapsulated (treated with bleach to remove the outer covering) to prevent any cross-contamination from surrounding water, or harvesting and processing steps. Any microbe rRNA gene sequences from decapsulated cysts would be more likely to be contained inside the cysts since, presumably, those on the outer covering would be removed by the bleach treatment during decapsulation.

## Conclusions

The primary goal of our study was to investigate the range of bacteria and archaea found in association with *Artemia* throughout its life cycle using 16S rRNA gene sequences isolated from adults and cysts. The difference in abundance of microbial sequences in our different adult *Artemia* samples supports the hypothesis that the character of the adult *Artemia* microenvironment may change in response to changes in temperature and salinity of GSL. It is important to note that while we did wash the surface of the animals in our samples, we did not dissect the adult gut or brood sack. Therefore, the microbial genes amplified from DNA extracted from adult *Artemia* could be from any of their internal regions or tightly associated with the exoskeleton. Also, our sampling efforts were limited to two sites at two different times. A more comprehensive set of samples that spans several sites and follows those sites for a number of seasons and years would provide a broader picture of these associations.

Sequences common to adults and GSL water may represent microbes that are used as food or microbes that were strongly attached to the external regions of adult *Artemia*. However, we did isolate adult specific microbial sequences, most notably AAC1 and ABC1. We are currently interested in investigating how these microbes are important to *Artemia* and whether they are in adult *Artemia* populations from other environments. We are also intrigued by the sequences shared by both adults and cysts (e.g. AAC3/GAC2) since it is possible, although untested so far, that they could be vertically transmitted through the *Artemia* germline. Alternatively, microbes may associate with *Artemia* every generation after birth or the brood sack may be permeable to the surrounding water. We did not detect the same microbes in water, although they may be present at very low abundance.

Encysted *Artemia* embryos also contained archaeal and bacterial sequences different from adults and GSL water. This discrepancy suggests that *Artemia* cysts may be a unique microenvironment that selects for microbes that can tolerate the special conditions inside cysts, such as desiccation. There are, however, some limitations to our current study. The cysts were commercially harvested, processed, and dried. This limits our knowledge of their true origin and our ability to compare their microbial communities.

The sequences that we have recovered from water, adults, and cysts support our hypothesis that *Artemia* harbors several microenvironments that contain microbes not abundant in the surrounding waters of GSL. The uniqueness of the microbe population in different stages of the life cycle of *Artemia* when compared to water suggests that there may be *Artemia* specific microbes, which may be important to the brine shrimp life cycle and the surrounding food web. While our study has shown what microbes are present in the *Artemia* microenvironment, the importance of each microbial species to the brine shrimp life cycle and surrounding food web remains to be tested, and a more systematic sample of water, cysts, and adults from different locations at different times is necessary to strengthen the geographical and seasonal differences that we have identified.

## Methods

### Sample collection

We collected the adult brine shrimp used in this study from the South Arm of GSL at a site just off the southern shore of GSL referred to as Black Rock (latitude 40.724933, longitude -112.227482) in September 2006 and at a site south of the railroad causeway referred to as DWR3 (latitude 41.16746, longitude -112.6696117) in May 2007. Water was collected both in September 2006 and May 2007 at Black Rock. Dry *Artemia* cysts stated to be of GSL origin (Premium Grade Brine Shrimp Egg, Lot P101, collected from the south arm of GSL) and from the San Francisco Bay (SFB) strain were purchased from Brine Shrimp Direct (Ogden, UT) in July of 2006, who states that they were treated with hypochlorite solution during processing. Adult *Artemia* and cysts were washed with 100% ethanol and rinsed with ddH_2_O using a Nytex filter to minimize surface contamination, then homogenized using a sterile pellet pestle. Cysts were not decapsulated to keep the treatment of the *Artemia* samples consistent. The collected lake water was centrifuged until a pellet was visible.

### Amplification of 16S rRNA genes

DNA was extracted from the homogenate or water pellet using the FastDNA spin kit for soil (MP Biomedicals) according to the manufacturer’s protocol. The same procedure was used to extract DNA from control bacterial strain *E. coli* (strain OP50, ATCC) and haloarchaeal strain “Halorubrum salsolis” (GSL isolate, BK Baxter unpublished). Some of the extracted DNA samples were ethanol precipitated which had no noticeable effect on polymerase chain reaction (PCR) efficiency. PCR was performed on template DNA using primers specific to either bacterial [49] or archaeal [50] 16S rRNA genes. The primers also contained a uracil and short tag sequence (underlined) required by the USER cloning procedure (New England Biolabs).

archaeal primers1HKU: 5^′^GGAGACAUATTCCGGTTGATCCTGCCGG 3^′^; H589RU: 5^′^GGGAAAGUAGCTACGGACGCTTTAGGC 3^′^bacterial primersEC16FU: 5^′^GGAGACAUAGAGTTTGATCATGGCTCAG 3^′^; EC16RU: 5^′^GGGAAAGUGGTTACCTTGTTACGACTT 3^′^

PCR reactions contained between 50-1000 ng of template DNA (50-250 ng resulted in amplification most consistently) and Taq DNA polymerase with dNTPs, buffer and magnesium according to the manufacturers’ protocols (Invitrogen or New England Biolabs) with a final volume of 20 μL. Negative control reactions in which no template DNA was added were performed for each primer set for every PCR experiment. We found that these controls were essential to detect DNA contamination in the reagents such as the polymerase.

PCR reactions were incubated at 94°C for 4 minutes then subjected to 36 cycles of 94°C for 1 minute, 50°C for 1 minute, and 72°C for 1 minute with a final elongation step of 72°C for 3 minutes. Gel electrophoresis using a 1.5% agarose gel containing SYBR safe (Invitrogen) was used to verify amplification of bacterial or archaeal DNA. Bands in experimental lanes were excised and purified using the QIAEX II DNA Purification from Agarose Gel kit (QIAGEN) according to the manufacturer’s protocol.

### Cloning and sequencing of 16S amplicons

Purified amplicons were cloned using the USER Friendly Cloning Kit (New England Biolabs) and transformed into competent *Escherichia coli* (New England Biolabs) according to the manufacturer’s protocols. Transformed cells were selected on ampicilin and screened using blue/white selection according to the USER kit protocol (New England Biolabs). Colonies with inserts were grown overnight at 37°C in LB medium with ampicillin, and plasmid DNA was prepared either using the QIAprep spin miniprep kit (Qiagen), or the MoBio Mini Plasmid Prep Kit (ISC Bioexpress) according to the manufacturer’s protocol. Plasmid DNA was then sequenced using the GenomeLab Dye Terminator Cycle Sequencing with Quick Start Kit and run on a CEQ8000 Genetic Analysis System according to the manufacturer’s protocol (Beckman Coulter). Primers used in sequencing reactions were, -47 primer supplied with the sequencing kit or M13 -47 primers (Operon). Some of the amplicons were sequenced using both the -47 sequencing primer and the forward primer provided with the USER cloning kit. All trimmed sequence reads longer than 200 basepairs that made up the contigs were submitted to the NCBI Genbank database. Accession numbers are shown in Tables 1 and 2, with the exception of SAU7 which was too short to be submitted to Genbank.

### Sequence analysis

The sequence traces from each experiment were base-called, trimmed of vector sequences, and trimmed of low quality bases using CodonCode Aligner (CodonCode Corporation). Then sequences from each environment (adults, GSL cysts, SFB cysts, or water) were assembled into contigs in Aligner using the default parameters. The sequences were then individually examined to ensure the accuracy of the base calls. These initial contigs were then unassembled one at a time and reassembled after increasing the stringency of the alignment (alignment – 98% minimum identity, assembly – 95% minimum identity) to maintain the separation of less related sequences.

All sequences were checked for chimerism with the Pintail program [51], classified using the RDP Classifier [52,53] and compared to known sequences in the NCBI nr nucleotide database using the NCBI BLASTn algorithm [54,55]. The align two sequences function (bl2seq) of BLAST at the NCBI was used for direct comparison of two sequences from our samples.

### Statistical analysis

To determine if the distribution of sequences isolated from *Artemia* adults and cysts were significantly different from the distribution observed in water, we constructed contingency tables and calculated the likelihood-ratio using the chi-square test function of SPSS 15.0 (SPSS Inc.). Sequences were only included in the analysis if they occurred 3 or more times in any one sample (e.g. GSL water) or were isolated from multiple samples. To determine if the distributions of sequences isolated from *Artemia* adults collected during the Fall of 2006 at Black Rock and Spring of 2007 at DWR3 were significantly different from each other we again used SPSS to calculate the likelihood-ratio. The percent abundance of a sequence was calculated by dividing the number of clones with that sequence in the sample being analyzed by the total number of clones from the sample (Figures 1 and 2). Only contigs that were constructed from five or more total clones were included in the analysis of variation in different samples. We only calculated percent abundance from sequences isolated multiple times (as described above) since it would be impossible to tell what the real percent abundance might be of a sequence identified zero or one time. We focused on the most abundant sequences since we felt that they best characterized our sample.

## Abbreviations

GSL: Great salt lake; SFB: San Francisco Bay.

## Competing interests

The authors declare that they have no competing interests.

## Authors’ contributions

MR helped conceive and design the experiments, collected samples, preformed most of the PCR and sequencing, assisted with sequence analysis, and participated in drafting the manuscript. BB helped conceive and design the experiments, provided technical assistance, and primer sequences. BA helped design the experiments, provided technical support for PCR and sequencing, led the sequence analysis, and drafted the manuscript. All authors read and approved the final manuscript.
